# 1-(3-Bromo-2-thien­yl)ethanone

**DOI:** 10.1107/S1600536810034677

**Published:** 2010-09-11

**Authors:** M. Mahendra, H. K. Vivek, S. L. Gaonkar, B. S. Priya, S. Nanjunda Swamy

**Affiliations:** a Department of Studies in Physics, University of Mysore, Manasagangotri, Mysore 570 006, India; b Department of Biotechnology, Sri Jayachamarajendra College of Engineering, Mysore 570 006, India; c Department of Studies in Chemistry, University of Mysore, Manasagangotri, Mysore 570 006, India

## Abstract

In the title compound, C_6_H_5_BrOS, the non-H and aromatic H atoms lie on a crystallographic mirror plane. In the crystal, mol­ecules are linked into chains propagating along the *c* axis by inter­molecular C—H⋯O hydrogen bonds.

## Related literature

For the uses of acetyl thio­phenes, see: Ashalatha *et al.* (2009 ▶); Bando *et al.* (2010 ▶); Ito & Furukawa (1990 ▶); Lutz *et al.* (2005 ▶); Nakayama *et al.* (1989 ▶); Pelly *et al.* (2005 ▶); Yasuhara *et al.* (2002 ▶).
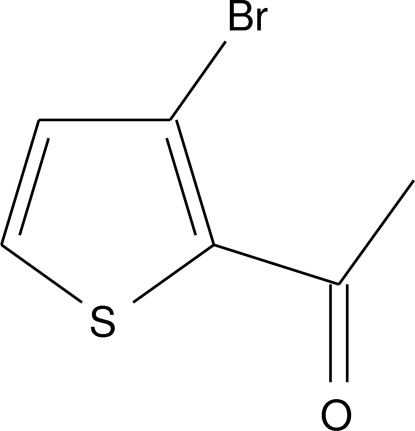


## Experimental

### 

#### Crystal data

C_6_H_5_BrOS
*M* *_r_* = 205.07
Orthorhombic, 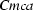

*a* = 6.8263 (17) Å
*b* = 13.149 (4) Å
*c* = 16.007 (4) Å
*V* = 1436.8 (7) Å^3^
*Z* = 8
Mo *K*α radiation
μ = 5.92 mm^−1^
*T* = 293 K
0.25 × 0.21 × 0.20 mm

#### Data collection

Bruker APEXII CCD area-detector diffractometer
Absorption correction: multi-scan (*SADABS*; Sheldrick, 2001 ▶) *T*_min_ = 0.313, *T*_max_ = 0.384
12363 measured reflections
973 independent reflections
790 reflections with *I* > 2σ(*I*)
*R*_int_ = 0.041

#### Refinement

*R*[*F* ^2^ > 2σ(*F* ^2^)] = 0.027
*wR*(*F* ^2^) = 0.067
*S* = 1.06
973 reflections
56 parameters
H-atom parameters constrained
Δρ_max_ = 0.68 e Å^−3^
Δρ_min_ = −0.48 e Å^−3^

### 

Data collection: *APEX2* (Bruker, 2004 ▶); cell refinement: *SAINT* (Bruker, 2004 ▶); data reduction: *SAINT*; program(s) used to solve structure: *SHELXS97* (Sheldrick, 2008 ▶); program(s) used to refine structure: *SHELXL97* (Sheldrick, 2008 ▶); molecular graphics: *PLATON* (Spek, 2009 ▶) and *ORTEP-3* (Farrugia, 1997 ▶); software used to prepare material for publication: *SHELXL97* and *PLATON*.

## Supplementary Material

Crystal structure: contains datablocks global, I. DOI: 10.1107/S1600536810034677/ci5166sup1.cif

Structure factors: contains datablocks I. DOI: 10.1107/S1600536810034677/ci5166Isup2.hkl

Additional supplementary materials:  crystallographic information; 3D view; checkCIF report

## Figures and Tables

**Table 1:** Hydrogen-bond geometry (Å, °)

*D*—H⋯*A*	*D*—H	H⋯*A*	*D*⋯*A*	*D*—H⋯*A*
C4—H4⋯O8^i^	0.93	2.43	3.352 (4)	174

Symmetry code: (i) 
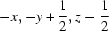
.

**Fig. 1.**
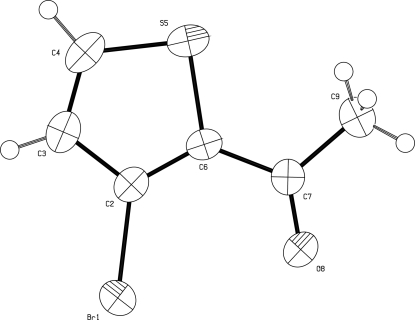
Molecular structure of (I), showing 50% probability displacement ellipsoids and the atomic numbering.

**Fig. 2.**
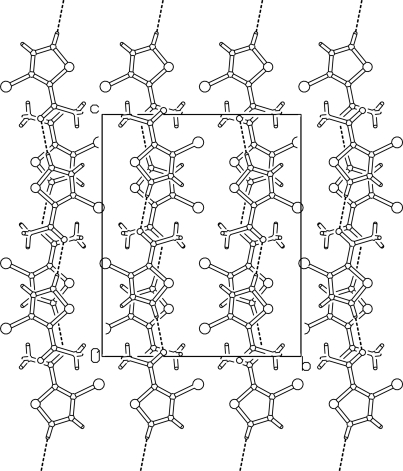
Packing diagram of (I), viewed down the a axis. The dashed lines represent hydrogen bonds.

## supplementary crystallographic information

### Comment

2-Acetyl-3-bromothiophene is one of the well-known bio-active intermediate used for the construction of number of new heterocycles (Lutz *et al.* 2005; Pelly *et al.* 2005). It is used as an intermediate for the synthesis of furo[3,2-a]carbazole alkaloid, furostifoline (Ito *et al.* 1990) and its derivatives, which show broad pharmacological properties (Yasuhara *et al.* 2002). Chalcones of 2-acetyl-3-bromothiophene exhibit promising anti-inflammatory, analgesic and antibacterial activities (Ashalatha *et al.* 2009). Acetyl thiophenes are useful as intermediates for preparing number of pharmaceutical compounds (Bando *et al.* 2010). Acetyl bromothiophenes are also used for the synthesis of number of biologically active pyridazine derivatives (Nakayama *et al.* 1989). With this background, the title compound (I), was synthesized and we report its crystal structure here.

The non-hydrogen and aromatic hydrogen atoms of the title molecule lie on a crystallographic mirror plane (Fig. 1). The molecules are linked into a chain along the *c* axis by intermolecular C—H···O hydrogen bonds (Table 1).

### Experimental

A three-necked, round-bottomed flask was charged with CH_2_Cl_2_ (10 ml) and anhydrous AlCl_3_ (2.45 g, 18.4 mmol). The flask was cooled to 273 K. A dropping funnel was charged with freshly distilled acetyl chloride (1.48 g, 19.6 mmol) in CH_2_Cl_2_ (15 ml), and was added drop wise for a period of 30 min. The reaction mixture was stirred for 1 h at 273 K. The reaction mass was further cooled to 250 K. 3-Bromothiophene (1.00 g, 6.13 mmol) in CH_2_Cl_2_ (15 mL) was added drop wise for 1 h. The reaction was stirred at 250 K for 30 min and then warmed slowly to room temperature and stirred for 1 h. Then the reaction mixture was quenched on ice water (50 ml). Layers were separated and aqueous layer was repeatedly extracted with CH_2_Cl_2_ and the combined organic extracts were washed with saturated NaHCO_3_ (25 ml), then brine (25 ml) and finally dried over anhydrous Na_2_SO_4_. Solvent was removed by distillation at atmospheric pressure. The remaining oily mass was distilled under high vacuum  (403 K at 10 mbar) to give a pale yellow oil which was crystallized in n-hexane to give 2-acetyl-3-bromothiophene (1.10 g, 88 %) as a yellow solid. Block-shaped yellow single crystals were obtained by slow evaporation of an n-hexane solution.

### Refinement

H atoms were placed at idealized positions and allowed to ride on their parent atoms with C–H distances in the range 0.93–0.96 Å and *U*_iso_(H) = 1.2-1.5*U*_eq_(carrier atom).

### Crystal data

**Table:**

C_6_H_5_BrOS	*F*(000) = 800
*M**_r_* = 205.07	*D*_x_ = 1.896 Mg m^−^^3^
Orthorhombic, *C**m**c**a*	Mo *K*α radiation, λ = 0.71073 Å
Hall symbol: -C 2bc 2	Cell parameters from 1982 reflections
*a* = 6.8263 (17) Å	θ = 2.5–28.4°
*b* = 13.149 (4) Å	µ = 5.92 mm^−^^1^
*c* = 16.007 (4) Å	*T* = 293 K
*V* = 1436.8 (7) Å^3^	Block, yellow
*Z* = 8	0.25 × 0.21 × 0.20 mm

### Data collection

**Table:**

Bruker APEXII CCD area-detector diffractometer	973 independent reflections
Radiation source: fine-focus sealed tube	790 reflections with *I* > 2σ(*I*)
graphite	*R*_int_ = 0.041
ω and φ scans	θ_max_ = 28.4°, θ_min_ = 2.5°
Absorption correction: multi-scan (*SADABS*; Sheldrick, 2001)	*h* = −9→9
*T*_min_ = 0.313, *T*_max_ = 0.384	*k* = −17→17
12363 measured reflections	*l* = −21→20

### Refinement

**Table:**

Refinement on *F*^2^	Primary atom site location: structure-invariant direct methods
Least-squares matrix: full	Secondary atom site location: difference Fourier map
*R*[*F*^2^ > 2σ(*F*^2^)] = 0.027	Hydrogen site location: inferred from neighbouring sites
*wR*(*F*^2^) = 0.067	H-atom parameters constrained
*S* = 1.06	*w* = 1/[σ^2^(*F*_o_^2^) + (0.0333*P*)^2^ + 1.3829*P*] where *P* = (*F*_o_^2^ + 2*F*_c_^2^)/3
973 reflections	(Δ/σ)_max_ = 0.001
56 parameters	Δρ_max_ = 0.68 e Å^−^^3^
0 restraints	Δρ_min_ = −0.48 e Å^−^^3^

### Special details

**Table:**

Refinement. Refinement on *F*^2^ for ALL reflections except those flagged by the user for potential systematic errors. Weighted *R*-factors *wR* and all goodnesses of fit *S* are based on *F*^2^, conventional *R*-factors *R* are based on *F*, with *F* set to zero for negative *F*^2^. The observed criterion of *F*^2^ > σ(*F*^2^) is used only for calculating -*R*-factor-obs *etc*. and is not relevant to the choice of reflections for refinement. *R*-factors based on *F*^2^ are statistically about twice as large as those based on *F*, and *R*-factors based on ALL data will be even larger.

### Fractional atomic coordinates and isotropic or equivalent isotropic displacement parameters (Å^2^)

**Table:**

	*x*	*y*	*z*	*U*_iso_*/*U*_eq_	Occ. (<1)
Br1	0.00000	0.01659 (3)	0.38478 (2)	0.0547 (1)	
S5	0.00000	0.33480 (6)	0.30339 (5)	0.0463 (3)	
O8	0.00000	0.19098 (19)	0.51639 (13)	0.0584 (9)	
C2	0.00000	0.1452 (2)	0.33378 (18)	0.0365 (9)	
C3	0.00000	0.1547 (3)	0.2458 (2)	0.0448 (10)	
C4	0.00000	0.2530 (3)	0.2207 (2)	0.0479 (10)	
C6	0.00000	0.2366 (2)	0.37465 (17)	0.0357 (9)	
C7	0.00000	0.2587 (2)	0.46527 (19)	0.0389 (9)	
C9	0.00000	0.3685 (3)	0.4922 (2)	0.0541 (11)	
H3	0.00000	0.09970	0.20930	0.0540*	
H4	0.00000	0.27350	0.16510	0.0570*	
H9A	0.13140	0.39450	0.49060	0.0810*	0.500
H9B	−0.05010	0.37360	0.54810	0.0810*	0.500
H9C	−0.08130	0.40740	0.45510	0.0810*	0.500

### Atomic displacement parameters (Å^2^)

**Table:**

	*U*^11^	*U*^22^	*U*^33^	*U*^12^	*U*^13^	*U*^23^
Br1	0.0910 (3)	0.0316 (2)	0.0415 (2)	0.0000	0.0000	−0.0009 (1)
S5	0.0590 (5)	0.0401 (4)	0.0397 (4)	0.0000	0.0000	0.0117 (3)
O8	0.106 (2)	0.0408 (13)	0.0285 (12)	0.0000	0.0000	0.0023 (10)
C2	0.0400 (16)	0.0399 (16)	0.0297 (14)	0.0000	0.0000	0.0021 (12)
C3	0.0512 (18)	0.0519 (18)	0.0314 (15)	0.0000	0.0000	−0.0035 (13)
C4	0.0526 (19)	0.063 (2)	0.0281 (14)	0.0000	0.0000	0.0069 (14)
C6	0.0428 (16)	0.0333 (14)	0.0309 (15)	0.0000	0.0000	0.0057 (11)
C7	0.0482 (17)	0.0354 (15)	0.0332 (15)	0.0000	0.0000	−0.0021 (12)
C9	0.079 (2)	0.0385 (16)	0.0448 (19)	0.0000	0.0000	−0.0061 (14)

### Geometric parameters (Å, °)

**Table:**

Br1—C2	1.878 (3)	C3—H3	0.93
S5—C4	1.706 (4)	C4—H4	0.93
S5—C6	1.723 (3)	C9—H9A	0.96
O8—C7	1.209 (4)	C9—H9B	0.96
C2—C3	1.414 (4)	C9—H9C	0.96
C2—C6	1.368 (4)	C9—H9A^i^	0.96
C3—C4	1.354 (6)	C9—H9B^i^	0.96
C6—C7	1.479 (4)	C9—H9C^i^	0.96
C7—C9	1.507 (5)		
			
Br1···O8	3.114 (3)	C3···C3^xi^	3.4158 (11)
Br1···Br1^ii^	3.7144 (12)	C3···C3^ix^	3.4158 (11)
Br1···O8^ii^	3.155 (3)	C3···C3^vi^	3.4158 (11)
Br1···S5^iii^	3.8453 (15)	C4···O8^xvi^	3.352 (4)
Br1···Br1^iv^	3.7144 (12)	C4···O8^xvii^	3.352 (4)
Br1···O8^iv^	3.155 (3)	C4···S5^x^	3.5993 (17)
Br1···S5^v^	3.8453 (15)	C4···S5^xi^	3.5993 (17)
S5···C4^vi^	3.5993 (17)	C4···C4^x^	3.5397 (16)
S5···Br1^vii^	3.8453 (15)	C4···C4^xi^	3.5397 (16)
S5···Br1^viii^	3.8453 (15)	C4···S5^ix^	3.5993 (17)
S5···C4^ix^	3.5993 (17)	C4···S5^vi^	3.5993 (17)
S5···C4^x^	3.5993 (17)	C4···C4^ix^	3.5397 (16)
S5···C4^xi^	3.5993 (17)	C4···C4^vi^	3.5397 (16)
S5···H9C^i^	2.6700	C7···C7^xiv^	3.5970 (17)
S5···H9C	2.6700	C7···C7^xviii^	3.5970 (17)
O8···Br1	3.114 (3)	C7···C7^xix^	3.5970 (17)
O8···C4^xii^	3.352 (4)	C7···C7^xv^	3.5970 (17)
O8···C4^xiii^	3.352 (4)	C9···C9^xx^	3.467 (6)
O8···Br1^ii^	3.155 (3)	C9···C9^xxi^	3.467 (6)
O8···Br1^iv^	3.155 (3)	H4···O8^xvi^	2.4300
O8···H4^xii^	2.4300	H4···O8^xvii^	2.4300
O8···H4^xiii^	2.4300	H9A···O8^xviii^	2.7600
O8···H9A^xiv^	2.7600	H9A···O8^xv^	2.7600
O8···H9A^xv^	2.7600	H9C···S5	2.6700
C3···C3^x^	3.4158 (11)		
			
C4—S5—C6	92.36 (16)	C7—C9—H9B	109.00
Br1—C2—C3	120.8 (2)	C7—C9—H9C	109.00
Br1—C2—C6	125.7 (2)	C7—C9—H9A^i^	109.00
C3—C2—C6	113.5 (3)	C7—C9—H9B^i^	109.00
C2—C3—C4	112.3 (3)	C7—C9—H9C^i^	109.00
S5—C4—C3	111.8 (2)	H9A—C9—H9B	109.00
S5—C6—C2	110.0 (2)	H9A—C9—H9C	109.00
S5—C6—C7	120.1 (2)	H9A—C9—H9A^i^	138.00
C2—C6—C7	129.9 (2)	H9A—C9—H9B^i^	71.00
O8—C7—C6	121.3 (3)	H9B—C9—H9C	109.00
O8—C7—C9	120.8 (3)	H9A^i^—C9—H9B	71.00
C6—C7—C9	118.0 (2)	H9B—C9—H9C^i^	138.00
C2—C3—H3	124.00	H9B^i^—C9—H9C	138.00
C4—C3—H3	124.00	H9C—C9—H9C^i^	71.00
S5—C4—H4	124.00	H9A^i^—C9—H9B^i^	109.00
C3—C4—H4	124.00	H9A^i^—C9—H9C^i^	109.00
C7—C9—H9A	109.00	H9B^i^—C9—H9C^i^	109.00
			
C6—S5—C4—C3	0.00	C3—C2—C6—S5	0.00
C4—S5—C6—C2	0.00	C3—C2—C6—C7	180.00
C4—S5—C6—C7	−180.00	C2—C3—C4—S5	0.00
Br1—C2—C3—C4	−180.00	S5—C6—C7—O8	−180.00
C6—C2—C3—C4	0.00	S5—C6—C7—C9	0.00
Br1—C2—C6—S5	180.00	C2—C6—C7—O8	0.00
Br1—C2—C6—C7	0.00	C2—C6—C7—C9	−180.00

Symmetry codes: (i) −*x*, *y*, *z*; (ii) *x*, −*y*, −*z*+1; (iii) −*x*, *y*−1/2, −*z*+1/2; (iv) −*x*, −*y*, −*z*+1; (v) *x*, *y*−1/2, −*z*+1/2; (vi) *x*+1/2, *y*, −*z*+1/2; (vii) −*x*, *y*+1/2, −*z*+1/2; (viii) *x*, *y*+1/2, −*z*+1/2; (ix) *x*−1/2, *y*, −*z*+1/2; (x) −*x*−1/2, *y*, −*z*+1/2; (xi) −*x*+1/2, *y*, −*z*+1/2; (xii) −*x*, −*y*+1/2, *z*+1/2; (xiii) *x*, −*y*+1/2, *z*+1/2; (xiv) *x*−1/2, −*y*+1/2, −*z*+1; (xv) −*x*+1/2, −*y*+1/2, −*z*+1; (xvi) −*x*, −*y*+1/2, *z*−1/2; (xvii) *x*, −*y*+1/2, *z*−1/2; (xviii) *x*+1/2, −*y*+1/2, −*z*+1; (xix) −*x*−1/2, −*y*+1/2, −*z*+1; (xx) *x*, −*y*+1, −*z*+1; (xxi) −*x*, −*y*+1, −*z*+1.

### Hydrogen-bond geometry (Å, °)

**Table:**

*D*—H···*A*	*D*—H	H···*A*	*D*···*A*	*D*—H···*A*
C4—H4···O8^xvi^	0.93	2.43	3.352 (4)	174

Symmetry codes: (xvi) −*x*, −*y*+1/2, *z*−1/2.
